# Angiotensin type-1 receptor autoantibodies promote alpha-synuclein aggregation in dopaminergic neurons

**DOI:** 10.3389/fimmu.2024.1457459

**Published:** 2024-11-11

**Authors:** Lucia Lage, Ana I. Rodríguez-Perez, Jose L. Labandeira-Garcia, Antonio Dominguez-Meijide

**Affiliations:** ^1^ Research Center for Molecular Medicine and Chronic diseases (CIMUS), IDIS, University of Santiago de Compostela, Santiago de Compostela, Spain; ^2^ Networking Research Center on Neurodegenerative Diseases (CIBERNED), Madrid, Spain

**Keywords:** alpha-synuclein, angiotensin, autoimmunity, neurodegeneration, Parkinson, protein aggregates

## Abstract

Angiotensin, through its type-1 receptor (AT1), is a major inducer of inflammation and oxidative stress, contributing to several diseases. Autoimmune processes have been involved in neurodegeneration, including Parkinson’s disease (PD). AT1 autoantibodies (AT1-AA) enhance neurodegeneration and PD, which was related to increased neuronal oxidative stress and neuroinflammation. However, the effect of AT1-AA on α-synuclein aggregation, a major factor in PD progression, has not been studied. In cultures of dopaminergic neurons, we observed that AT1-AA promote aggregation of α-synuclein, as AT1-AA upregulated major mechanisms involved in the α-synuclein aggregation process such as NADPH-oxidase activation and intracellular calcium raising. The results further support the role of AT1 receptors in dopaminergic neuron degeneration, and several recent clinical studies observing the neuroprotective effects of AT1 receptor blockers.

## 1 Introduction

The peptide angiotensin II (Ang II), through its type-1 (AT1) receptor, is a major inducer of inflammation and oxidative stress in most tissues (1) and plays a major role in several diseases, including neurodegenerative diseases such as Parkinson’s disease (PD) (2). This latter is supported by recent studies in humans showing that the dopaminergic neurons most vulnerable to degeneration are characterized by high levels of AT1 gene expression (3). The role of autoimmune processes in neurodegeneration, including PD, has been recently revealed (4). Autoantibodies targeting extracellular domains of neuronal or glial proteins have been associated with several brain diseases including neurodegenerative diseases [see (5, 6) for review]. More than 25 different types of autoantibodies have been observed in patients with neurological diseases (5). Interestingly, autoantibodies for AT1 receptors (AT1-AA) have been involved in several peripheral inflammation-related diseases (7) including Covid-19 (8). It is known that AT1-AA act as AT1 receptor agonists, immediately and persistently activating these receptors (7, 8). We have recently observed an increase in AT1-AA expression in serum and cerebrospinal fluid in PD patients and PD experimental models, and that AT1-AA can affect dopaminergic neurons and disrupt the blood-brain barrier (BBB) (9). Cytokines generated during the neuroinflammation associated with the neurodegenerative process modify AT1 receptors thus generating neoantigens [(10); see Discussion], which can be released by the affected cells, particularly after neuron degeneration. Consistent with this, AT1-AA generated due to peripheral inflammation-related processes such as the metabolic syndrome affected the progression of dopaminergic neuron degeneration, revealing a new link factor between peripheral inflammation and PD (11). In PD, the production of AT1-AA may contribute to disease progression during the long period of active neuron death and neuroinflammation. In experimental models, we observed that AT1-AA enhanced the progression of dopaminergic degeneration (9), which was related to the increase in neuronal oxidative stress and neuroinflammation, two major factors involved in dopaminergic neuron death (9, 11). In addition, AT1-AA promote the disruption of the BBB (9, 11, 12), which may also contribute to neuroinflammation and neurodegeneration. It is known that aggregation and accumulation of α-synuclein is a major factor involved in the progression of dopaminergic degeneration and neuroinflammation. PD is characterized by the presence of protein aggregates termed Lewy bodies (LBs), and one of the characteristics of LBs is the presence of misfolded forms of α-synuclein (13). Aggregated misfolded α-synuclein spreads through the brain, propagating PD (14). However, the possible effect of AT1-AA on α-synuclein aggregation has not been studied. In the present experiments, we studied this question in cultures of dopaminergic neurons and the possible mechanisms involved in the observed effects.

## 2 Methods

### 2.1 Experimental design

Three main experiments were designed to study the effects of AT1-AA on α-synuclein aggregation in dopaminergic neurons and the main mechanisms that lead to these effects. First, we studied the effects of AT1-AA on α-synuclein aggregation using several different experimental groups: untreated control N27 dopaminergic neurons, N27 neurons treated with AT1-AA, and N27 neurons treated with AT1-AA and the AT1 blocker losartan. Finally, an additional control group consisted of cells treated with an “indifferent” antibody (i.e. non-target specific for neurons; anti-GFAP, Glial Fibrillary Acidic Protein). N27 is a neuronal rat dopaminergic cell line that we have already used in previous studies on the effects of AT1-AA (9). N27 cells showed responses to AT1-AA, which were similar to those observed in primary dopaminergic neurons (9). The presence of aggregates was assessed in the α-synuclein-T/V5-synphilin-1 (SynT/Sph1) aggregation model (15, 16). The suitability of N27 for use with the synuclein aggregation model used in the present study was confirmed in preliminary experiments. Second, as it is known that AngII/AT1 activation promotes intracellular calcium raising (17), we checked whether AT1-AA can also induce calcium raising. Third, we checked the effects that AT1-AA may exert on NADPH-oxidase activation, as AngII/AT1 activation increases NADPH-oxidase activity (18), and previous studies have shown that NADPH-oxidase-derived oxidative stress and calcium raising promote α-synuclein aggregation (see Discussion).

### 2.2 AT1-AA purification

AT1-AA were obtained from the serum of pregnant women who suffered preeclampsia. These women were patients from the Obstetric Service of the University Hospital Complex of Santiago de Compostela. All patients signed an informed consent and were informed of the study’s purpose and protocol. Approval for this study was obtained from the Galician Drug Research Ethics Committee (CEIm-G) on January 23, 2018, protocol 2017/618, and the research was carried out following the principles of the Helsinki Declaration. Isolation of AT1-AA from the serum samples was carried out by affinity chromatography and subsequent titration as detailed elsewhere (11). Anti-human IgG was purified by passing one ml of serum through a specific purification column. In brief, AT1-AA were separated from the human IgG fraction by binding the epitope to the corresponding sequence for the second extracellular loop of the AT1 receptor (SC1208; Chemical Peptide Synthesis) covalently bound to the gel activated by Sepharose 4B CNBr (GE17-0430-01; Sigma-Aldrich). Bound IgG was eluted with 200 mM glycine buffer after washing away the unbound IgG. Eluted AT1-AA were concentrated, and the buffer was exchanged into PBS using a 3 kDa Amicon Ultra centrifugal filter (Millipore, USA). The final concentration of AT1-AA was quantified in a Nanodrop Spectrophotometer 252 (Thermo Fisher Scientific). AT1-AA were added to the cells at a final concentration of 0.1 µg/µl. All the experiments were conducted using the same batch of autoantibodies, obtained from a pool of serum samples from preeclamptic patients.

### 2.3 Transfection of cell cultures

N27 rat dopaminergic neuron cells were cultured in Roswell Park Memorial Institute (RPMI) medium (Sigma-Aldrich, ref R8758-1640) supplemented with 10% FBS, 1% penicillin/streptomycin and 1% fungizone and kept at 37°C and 5% CO_2_ in a humidified incubator. Approximately 100,000 cells were plated per well in a 12-well plate (Falcon, ref 353043) and twenty-four hours later they were transfected on the plate with TRANSIT-X2 transfection reagent (Mirus Bio, ref 6004) according to the following protocol: 2 µl of TRANSIT-X2 and 1 µg of total DNA (1:1 SynT : Sph1) were added to 100 µl of Dulbecco’s modified Eagle medium (DMEM) medium (10-013-CV, Falcon Corning) and incubated for 20 min at room temperature in the dark. The resulting mixture was added dropwise to the cells, and the plate was gently rocked. Cells were kept in an incubator at 37°C and 5% CO_2_ for an additional 48 h. The plasmids used were α-Synuclein-T and V5-synphilin-1. These plasmids were cloned in PCDNA3.1+ mammalian vectors. Original plasmids were kindly donated by Dr. Tiago Outeiro, and new clones were prepared following published protocols (19). Overall transfection efficiency was determined at 41.5 ± 6.7% (Mean + SEM) by microscopical analysis.

### 2.4 Treatment of cell cultures

Forty-eight hours after transfection cells were fed with fresh medium, and 10 µM of losartan potassium (Sigma-Aldrich, ref 61188-100MG) was added dropwise. One hour later, the cells were treated with 0.1 µg/µl of AT1-AA. Additionally, as a positive control for cell death, a group of cells was treated with 20 µM of freshly prepared 6-OHDA (Sigma-Aldrich, ref H4381). Twenty-four hours after treatment, the cells were processed for immunofluorescence and NADPH-oxidase activity (Calcium imaging analysis was initiated just before AT1-AA treatment; see below). An additional control group of cultures was treated with primary antibodies against GFAP (0.1 µg/µl; Dako, ref Z0334) using the same protocol as AT1-AA.

### 2.5 Immunofluorescence of cell cultures

Cells transfected with α-Synuclein-T and V5-synphilin-1 were washed with Dulbecco’s phosphate-buffered saline (DPBS; pH 7.4) and fixed with 4% paraformaldehyde (PFA) for 20 min. Following fixation, a blocking and permeabilization step was performed for 1 h at room temperature with 1% BSA, 2% normal donkey serum (NDS, Sigma-Aldrich, ref D9663), and 1.5% Triton-X-100 in DPBS. Cells were then incubated with an anti-α-synuclein antibody MJFR1 (1:1500; Abcam, Rabbit, ref ab138501), in DPBS with 1% BSA and 2% NDS overnight at 4°C. After incubation with the primary antibody, cells were rinsed with DPBS and then incubated with the fluorescent secondary antibody Alexa Fluor 488-conjugated donkey anti-rabbit IgG (1:200; Thermo Fisher Scientific, ref A-21206) for 2 h. Finally, cells were incubated for 10 min with the DNA-binding dye Hoechst 33342 (1:8000 in DPBS; ref H1399; Thermo Fisher Scientific), washed three times with DPBS, and kept until analysis.

### 2.6 Inclusion quantification

For inclusion quantification, pictures were taken on a Leica DMI6000B fluorescence microscope at x20 with an HC PL Fluotar L x20/0.40 dry objective (reference 11506242), or at x40 with an HC PL Fluotar L ×40/0.60 dry objective (reference 11506201), and a DFC360FX-31202708 camera with 460, and 527 nm filters simultaneously. A minimum of 30 pictures were taken for each treatment and experiment. Pictures were visualized in ImageJ (https://imagej.nih.gov/ij/), and inclusions were visually identified in every picture and manually counted for each transfected cell.

### 2.7 NADPH-oxidase activity measurements

The cells were collected and homogenized in krebs-hepes buffer. After homogenization, protein concentration of the resultant suspension was measured using the Pierce BCA Protein Assay Kit (Thermo Fisher Scientific, ref 23225). 30 μg of extracted proteins were loaded into white 96-well plates, and NADPH-oxidase activity was measured in the presence of lucigenin (5 × 10^−6^ mol/L; Sigma-Aldrich, ref M8010) and NADPH (10− 4 mol/L; Sigma-Aldrich, ref N1630). Measurements were performed with an Infinite M200 multiwell plate reader (Tecan, Switzerland).

### 2.8 Intracellular calcium measurements

Changes in intracellular Ca^2+^ were estimated using the Fluo-4 AM Ca^2+^ sensor (Thermo Fisher Scientific). Cells treated and not treated with losartan were immediately incubated in 5 µM Fluo-4 AM at 37°C for 60 min. Cells were then washed with calcium-free buffer, and cell fluorescence was analyzed using a widefield microscope (Leica DMI6000B) (488 nm excitation, 520 nm emission) equipped with a chamber with CO_2_, humidity, and temperature control. AT1-AA treatment was performed during live-cell imaging. Each cell was defined as a region of interest (ROI) and their fluorescence intensity was subsequently measured. The changes in Fluo-4 AM fluorescence intensity correlate with intracellular Ca^2+^ levels. These changes in fluorescence intensity were measured as normalized fluorescence (ΔF) relative to basal fluorescence (recording period before AT1-AA addition).

### 2.9 Statistical analysis

The normality of the data distributions and the homogeneity of variances were assessed before each analysis of variance (ANOVA) using the Kolmogorov–Smirnov test. For experiments involving a single factor, one-way ANOVA was employed, using the Student-Newman-Keuls method as *post hoc* test. Two-way ANOVA or the corresponding non-parametric alternative was utilized for studies with two factors, with Bonferroni as *post hoc* analysis to evaluate pairwise differences. Statistical significance was determined at a threshold of P ≤ 0.05. Results are presented as mean values ± standard error of the mean (SEM), derived from at least six independent experiments. All statistical analyses were performed using SigmaPlot 11.0 (Systat Software, Inc., CA, U.S.A.). GraphPad Prism 8 software (GraphPad; Inc., San Diego, CA, USA) was used to create scatter dot plot graphs.

## 3 Results

### 3.1 AT1 autoantibodies promote α-synuclein aggregation

**Figure 1:**
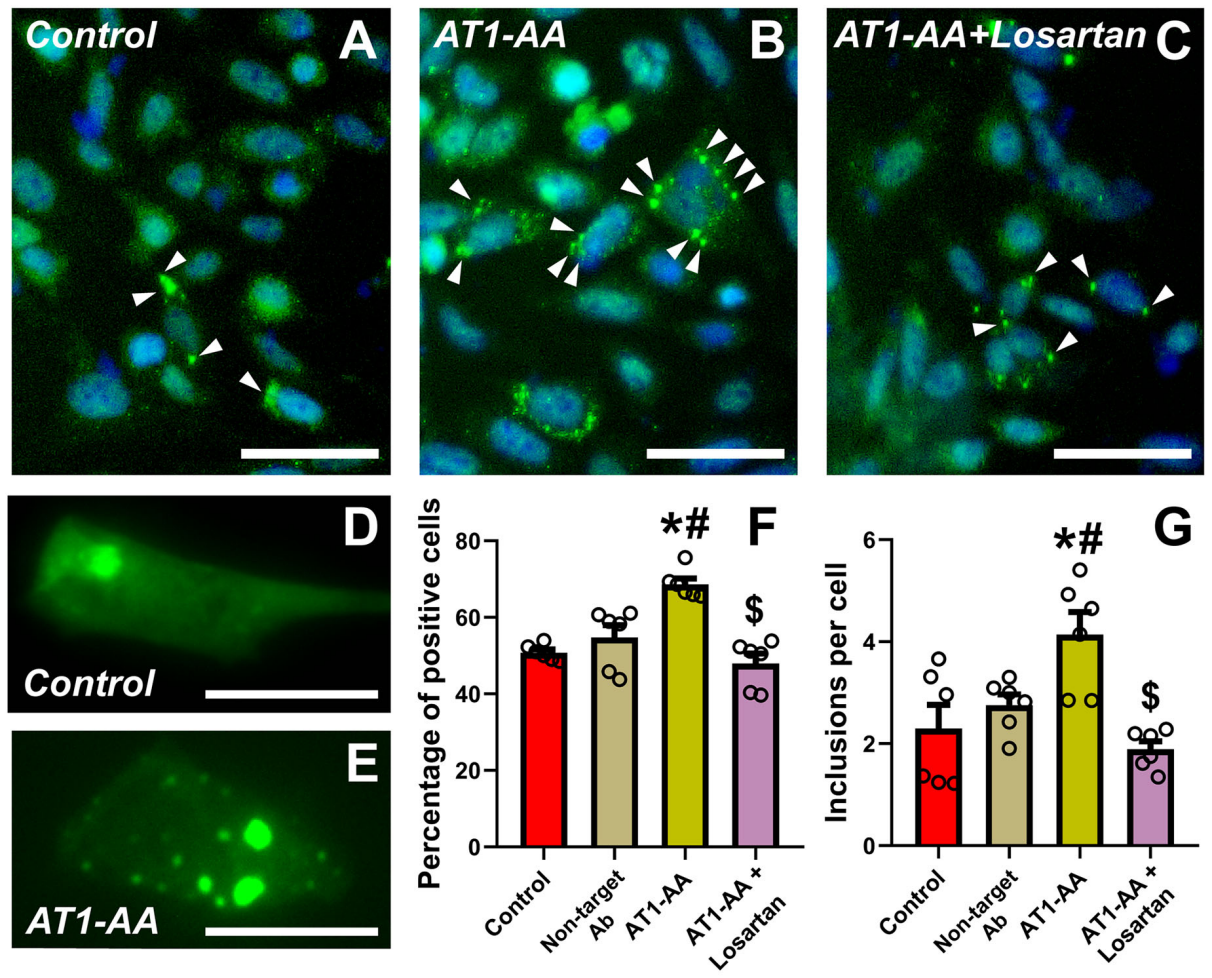
Treatment with AT1-AA increases α-synuclein aggregation. **(A–E)** Representative photographs at low **(A–C)** and high **(D, E)** magnification of N27 dopaminergic neurons showing α-synuclein inclusions (arrowheads). Scale bars: 50 µm **(A–C)** and 15 µm **(D, E)**. **(F)** Percentage of transfected cells showing inclusions. Treatment with AT1-AA significantly increases the percentage of cells showing inclusions. Treatment with losartan blocks the effects of the AT1-AA **(A–C, F)**. **(G)** Average number of inclusions per cell. Treatment with AT1-AA significantly increases the average number of inclusions per cell. This increase is blocked by treatment with losartan **(A–E, G)**. n =6. *P < 0.05 related to the control group, #P < 0.05 compared to non-target Ab group; $ P < 0.05 compared to AT1-AA group. One-way ANOVA **(F)** and Kruskal-Wallis One Way Analysis of Variance on Ranks **(G)** with Student-Newman-Keuls method *post hoc* test. Error bars represent SEM. Ab, Antibody; AT1-AA, Autoantibodies for AT1 receptors.

**Figure 2:**
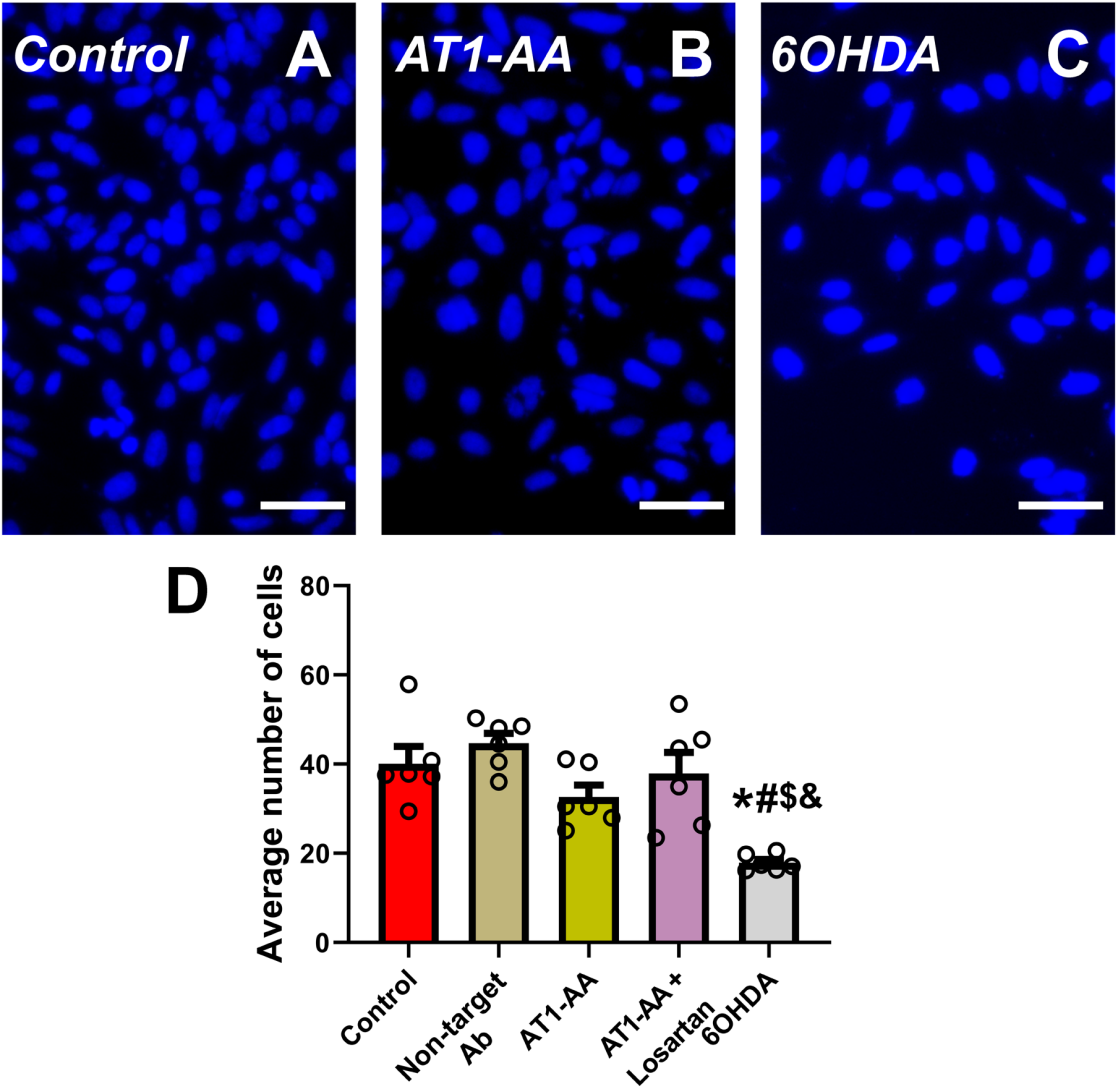
Average number of cells per microscopic field **(A–D)**. In the present model, treatment with AT1-AA alone induced a small non-significant decrease in the number of dopaminergic neurons. Treatment with low doses of the dopaminergic neurotoxin 6-OHDA (20 µM) significantly decreased the number of cells and was used as positive control. Neurons were identified and counted using Hoechst staining **(A–C)**; n =6. *P < 0.05 related to the control group, #P < 0.05 compared to non-target Ab group; $ P < 0.05 compared to AT1-AA group, & P < 0.05 compared to AT1-AA+Losartan group. One-way ANOVA with Student-Newman-Keuls method *post hoc* test. Scale bars: 50 µm. Error bars represent SEM. Ab: Antibody; AT1-AA: Autoantibodies for AT1 receptors.

Our results show that the number of transfected N27 dopaminergic neurons with α-synuclein inclusions significantly increased after treatment of cultures with AT1-AA. Treatment of cultures with the AT1 receptor blocker losartan inhibited the effects of AT1-AA, indicating that those effects were mediated by AT1 receptor overactivation (**Figures 1A–F**). Furthermore, treatment with AT1-AA led to a significant increase in the average number of inclusions per cell. Again, this effect was inhibited by the AT1 blocker losartan (**Figures 1A–E, G**). Consistent with our observations in previous studies (9, 11) these treatments with AT1-AA, in the absence of other deleterious factors, did not produce a statistically significant reduction in the number of dopaminergic neurons (**Figures 2A–D**). This was measured as the number of cells in a series of at least 180 pictures analyzed over 6 different experiments. As a positive control for cell death, we used 6-OHDA, which led to a significant decrease in the number of cells observed (**Figures 2C, D**). These results confirmed that AT1-AA treatment increases α-synuclein aggregation, which needs to act synergistically with other factors to induce significant neuron death, at least in the present experimental conditions. Effects induced by AT1-AA were not observed when a non-target antibody, “indifferent” for neurons, (anti-GFAP) was used (**Figures 1F, G**, **2D**).

### 3.2 Intracellular calcium raising and NADPH-oxidase activation mediate AT1-AA-induced increase in α-synuclein aggregation

**Figure 3:**
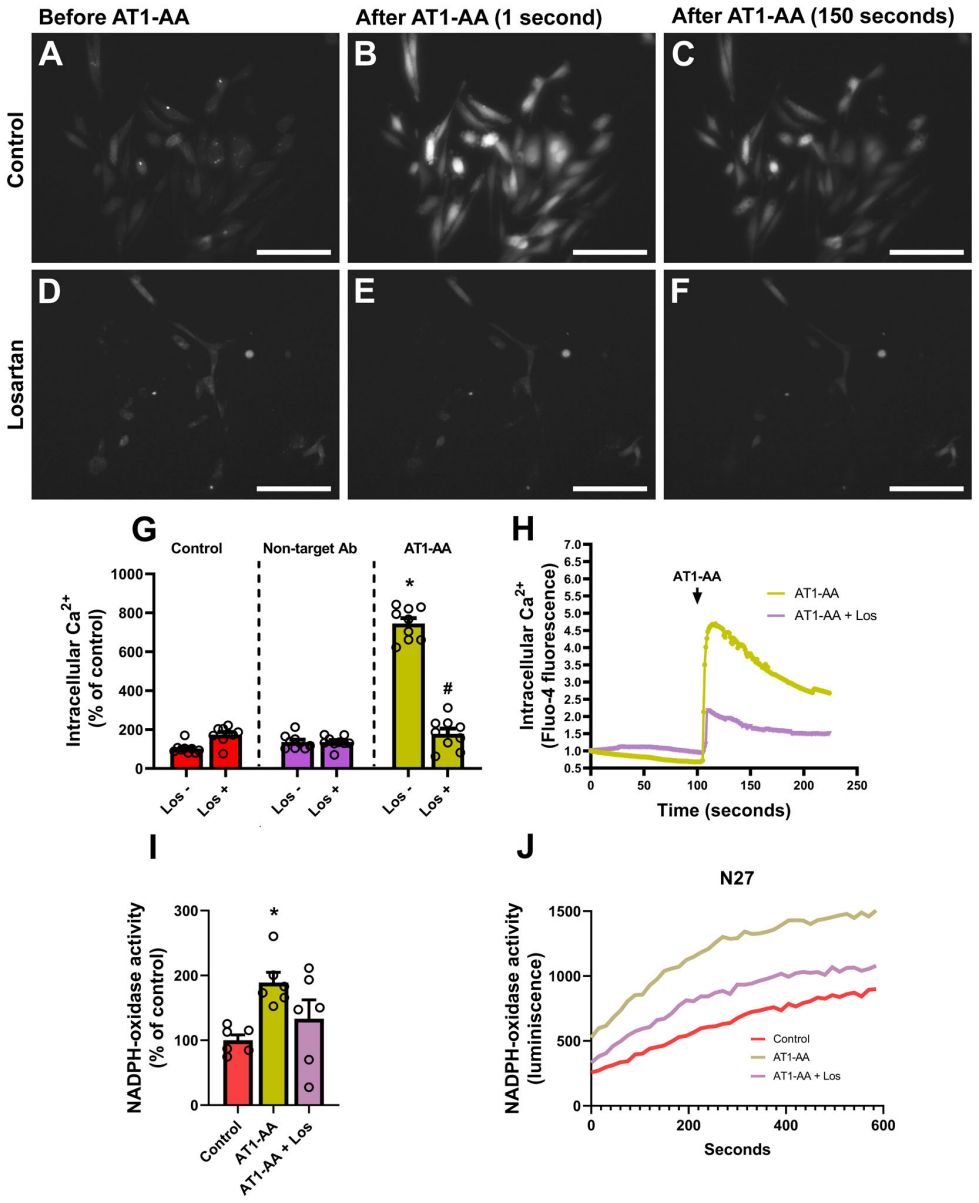
AT1-AA-mediated changes in calcium levels and NADPH-oxidase activity. **(A–C)** Representative wide-field pictures of live cells treated with Fluo-4 AM showing an increase in fluorescence after the addition of AT1-AA. Scale bars: 50 µm. **(D–F)** Representative wide-field pictures of live cells treated with Fluo-4 AM and the AT1 blocker losartan showing that AT1-AA did not induce a significant increase in fluorescence in the presence of losartan. Scale bars: 50 µm. **(G, H)** Treatment with losartan inhibited the effect of AT1-AA on intracellular calcium release; no significant changes were observed in control cells treated with losartan or in cells treated with a non-target antibody, either in the presence or the absence of losartan. **(I, J)** Treatment with losartan decreased the effect of AT1-AA on NADPH-oxidase activation; n = 6. * P< 0.05 compared with the control groups (with/without losartan) and control cells treated with a non-target antibody (with/without losartan) in G, and compared with the corresponding control in I. # P < 0.05 compared with AT1-AA. Multiple comparisons **(G)** were analyzed by two-way ANOVA on ranks Kruskal-Wallis with Bonferroni method as *post hoc* test. Simple comparisons **(I)** were analyzed by one-way ANOVA with Student-Newman-Keuls method *post hoc* test. Error bars represent SEM. Ab, Antibody; AT1-AA, Autoantibodies for AT1 receptors; Los, Losartan.

In previous studies, oxidative stress induced by NADPH-derived superoxide and the increase in intracellular calcium levels have been involved in α-synuclein aggregation, and both factors may be caused by AT1 activation (see Discussion). We estimated intracellular calcium levels using a fluorescent commercial sensor that increases its light emission whenever intracellular calcium levels increase (**Figures 3A–C**). We observed a marked increase in fluorescence (i.e. intracellular calcium levels) after treatment with AT1-AA, which was inhibited by treatment with the AT1 blocker losartan (**Figures 3A–H**). Additionally, treatment with AT1-AA led to an increase in NADPH-oxidase activity, which was decreased by treatment with the AT1 blocker losartan (**Figures 3I, J**). The results indicate that AT1-AA increase both intracellular calcium levels and NADPH-oxidase activity, which can enhance α-synuclein aggregation.

## 4 Discussion

Autoantibodies targeting extracellular domains of neuronal or glial proteins have been associated with several brain diseases, including neurodegenerative diseases (5). Autoantibodies for G Protein-Coupled Receptors (GPCRs) can play agonist-stimulatory or antagonist-inhibitory effects on GPCRs and induce functional cell dysregulations that may promote diseases (6). Circulating autoantibodies may enter cerebrospinal fluid and the brain by crossing a disrupted BBB, although their intrathecal synthesis by activated B cells that migrated into the intrathecal compartment has also been shown (5, 20). The mechanisms responsible for the different autoantibody generation are unclear, including possible defects in the immune checkpoints and other possible mechanisms (5, 6). In the case of AT1-AA, inflammation-related cytokines have been shown to modify AT1 receptors, transforming them into neoantigens responsible for the induction of AT1-AA (11, 21). Infusion of IL-6 and TNF-α in animal models led to increased levels of AT1-AA (22, 23). However, TNFSF14 (LIGHT), through transglutaminase 2 (TG2) induction, plays a major role (10). TG2 modifies AT1 receptors (15, 24, 25) and generates neoantigens that lead to AT1-AA generation (24). These neoantigens may be released by cells and particularly by dopaminergic neurons during de neurodegenerative process. Consistent with this mechanism, we observed a significant correlation between AT1-AA and LIGHT levels in COVID-19 patients (8), and animal models and patients of PD (9, 11).

It has been suggested that AT1-AA may act as potent activators of AT1 receptors because, in addition to their agonist effect, AT1-AA binding to AT1 receptors stabilizes the receptor in a permanently activated state by inhibiting their internalization thus upregulating AT1 receptor expression (25). Proinflammatory-prooxidative AT1 receptors have been observed in dopaminergic neurons and glial cells, contributing to regulating neuronal oxidative stress and microglial pro-inflammatory responses (3, 18). Using confocal microscopy and fluorescent labelling of AT1-AA, we showed binding of AT1-AA to dopaminergic neuron and glial cell surfaces, presumably to AT1 receptors (9). Furthermore, administration of AT1-AA to cultures enhanced proinflammatory cytokine levels and dopaminergic neuron death, which was inhibited by simultaneous treatment with AT1 antagonists (9). Interestingly, several studies have shown that activation of endothelial AT1 receptors induces disruption of BBB (12), and we observed that intraperitoneal chronic infusion of AT1-AA also induced BBB disruption (11) which may act as an additional factor enhancing neurodegeneration.

**Figure 4:**
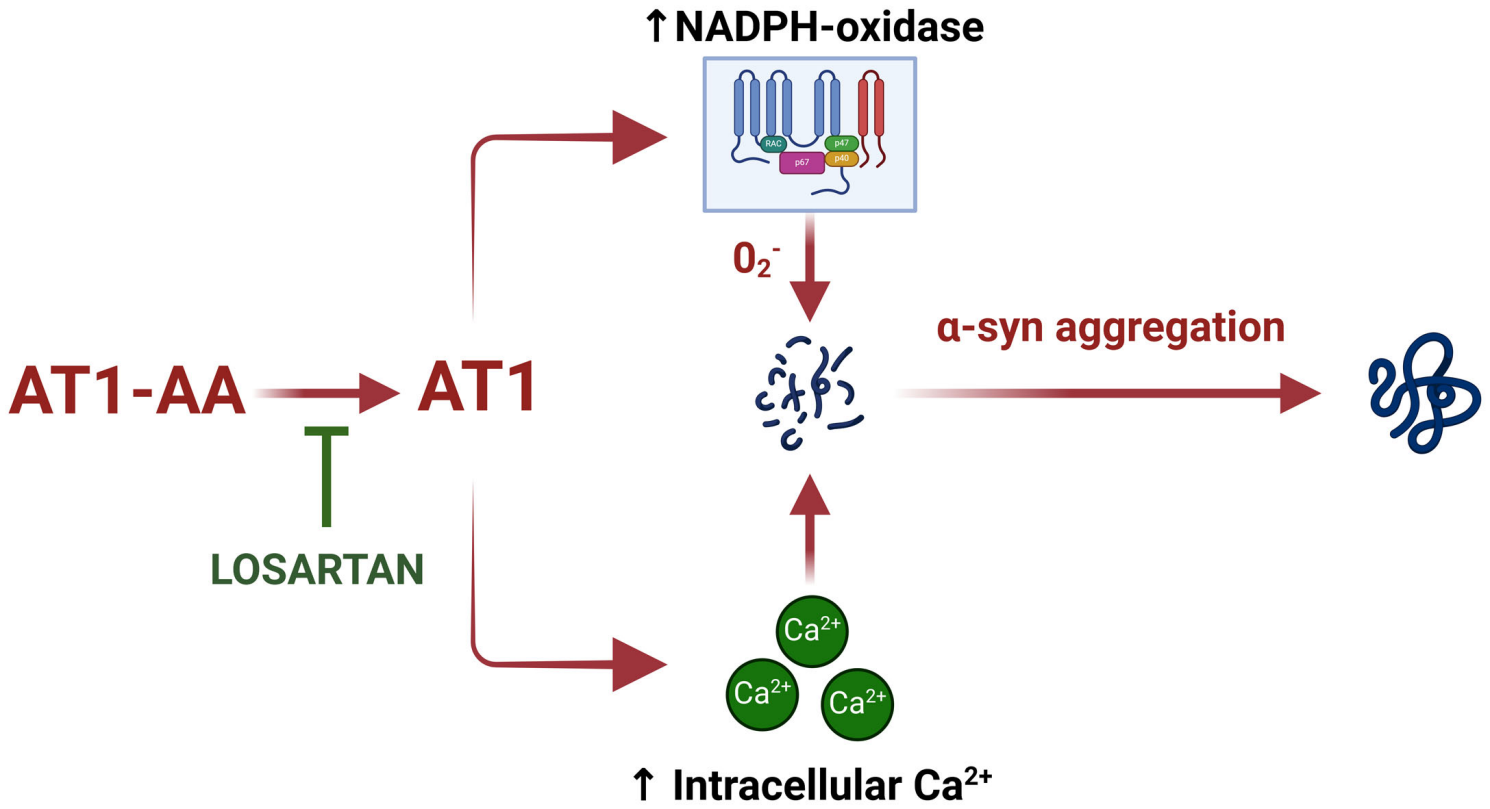
Schematic model summarizing the effect of AT1-AA on aggregation of α-synuclein in dopaminergic neurons. AT1-AA promote α-synuclein aggregation by upregulating NADPH-oxidase activation and intracellular calcium raising. This figure was created using BioRender.com under a licensed agreement.

In the present study, we observed that AT1-AA can contribute to dopaminergic degeneration by promoting aggregation of α-synuclein in dopaminergic neurons, as AT1-AA can upregulate major mechanisms involved in the aggregation process such as NADPH-oxidase activation and intracellular calcium raising (**Figure 4**). Previous studies have shown that the major consequences of AT1 receptor overactivation are the NADPH-oxidase-derived oxidative stress and intracellular calcium mobilization (2). It is known that NADPH-oxidase-derived superoxide enhances α-synuclein aggregation into oligomers (26). Intracellular free calcium raising also increases α-synuclein aggregation, and synergistic effects of calcium and oxidative stress further enhance α-synuclein aggregation, as it was observed that calcium raising alone leads to non-stable aggregates, while calcium plus oxidative stress produces more stable and larger aggregates (27). In addition, we confirmed that the enhancing effect of AT1-AA on α-synuclein aggregation is produced through AT1 receptor activation because it was inhibited by the AT1 blocker losartan. As observed in our previous studies using low doses of angiotensin and neurotoxins such as 6-OHDA or MPTP (18, 28), or AT1-AA at similar doses (9), the present doses of AT1-AA alone did not induce a significant loss of dopaminergic neurons but may synergistically enhance the effect of other deleterious factors such as low doses of dopaminergic neurotoxins like 6-OHDA (9). This is consistent with the multifactorial origin and the multiple-hit hypothesis for PD (29). In addition, dopaminergic neurodegeneration and neuroinflammation related to PD further induce AT1-AA production (9), leading to a vicious cycle. In conclusion, the results further support the major role of AT1 receptors in dopaminergic degeneration and PD, where the most vulnerable neurons have the highest expression of the AT1 receptor gene (3), and support several recent clinical studies (30) observing the neuroprotective effects of AT1 receptor blockers (i.e. sartans) on PD risk and progression. The present study shows that the presence of agonistic AT1-AA promotes alpha-synuclein oligomers and higher-level aggregates, which is a major mechanism of PD progression.

## Data Availability

The raw data supporting the conclusions of this article will be made available by the authors, without undue reservation.
